# Phenotypic Susceptibility, Resistance-Associated Genomic Determinants, and Mobile-Genetic-Element Context of Canine Otitis Externa-Associated *Pseudomonas aeruginosa* Collected in Hungary in 2010 and 2017

**DOI:** 10.3390/vetsci13090855

**Published:** 2026-08-23

**Authors:** Mercédesz Adrienn Veres, Zsófia Anna Tóth, Enikő Illés, Patrik Mag, Eszter Kaszab, Enikő Fehér, Ákos Jerzsele, Ádám Kerek

**Affiliations:** 1 Department of Pharmacology and Toxicology, University of Veterinary Medicine Budapest, István Utca 2, H-1078 Budapest, Hungary; veres.a.mercedesz@univet.hu (M.A.V.); toth.zsofia.anna@student.univet.hu (Z.A.T.); illes.eniko@student.univet.hu (E.I.); mag.patrik@univet.hu (P.M.); jerzsele.akos@univet.hu (Á.J.); 2 National Laboratory of Infectious Animal Diseases, Antimicrobial Resistance, Veterinary Public Health and Food Chain Safety, University of Veterinary Medicine Budapest, István Utca 2, H-1078 Budapest, Hungary; kaszab.eszter@univet.hu (E.K.); feher.eniko@univet.hu (E.F.); 3 Department of Bioinformatics, One Health Institute, Faculty of Health Sciences, University of Debrecen, Nagyerdei Krt. 98, H-4032 Debrecen, Hungary; 4 Department of Microbiology and Infectious Diseases, University of Veterinary Medicine Budapest, István Utca 2, H-1078 Budapest, Hungary; 5 National Laboratory of Virology, Szentágothai Research Centre, University of Pécs, Ifjúság Útja 20, H-7624 Pécs, Hungary

**Keywords:** antimicrobial resistance, canine otitis externa, chlorhexidine, fluoroquinolones, minimum inhibitory concentration, mobile genetic elements, *Pseudomonas aeruginosa*, resistance determinants, whole-genome sequencing

## Abstract

**Background:** Canine otitis externa caused by *Pseudomonas aeruginosa* is frequently chronic and treatment refractory, yet integrated historical phenotype–genotype data remain limited. We characterized an archival Hungarian collection obtained in 2010 and 2017. **Methods:** Broth microdilution minimum inhibitory concentrations (MICs) were determined for 67 isolates against 13 antimicrobial agents and chlorhexidine, and 59 isolates had matched long-read whole-genome assemblies. Resistance-associated determinants were identified using the Comprehensive Antibiotic Resistance Database, and their predicted proximity to mobile genetic elements and plasmid-like contigs was evaluated. **Results:** Current canine Clinical and Laboratory Standards Institute breakpoints classified 59/67 isolates (88.1%) as enrofloxacin resistant and 43/67 (64.2%) as marbofloxacin resistant. European Committee on Antimicrobial Susceptibility Testing epidemiological cut-off values identified non-wild-type subsets for ciprofloxacin (7/67), tobramycin (4/67), imipenem (1/67), and piperacillin–tazobactam (51/67), whereas all isolates remained within the available wild-type distributions for ceftazidime, gentamicin, and amikacin. No continuous MIC distribution changed significantly between sampling years after false-discovery-rate correction, although categorical marbofloxacin resistance decreased from 87.5% in 2010 to 42.9% in 2017. Resistance-gene screening identified 64 unique determinants, including near-universal efflux-system components, PDC and OXA-50-like β-lactamases, *APH(3′)-IIb*, *arnA*, and *basS*. The fluoroquinolone-associated determinant *crpP* occurred in 37/59 genomes and was located on plasmid-predicted contigs in six isolates. No determinant–MIC association remained significant after correction for multiple testing. **Conclusions:** The discordance between conserved resistance-gene repertoires and heterogeneous MICs demonstrates that sequence-based detection alone is insufficient for therapeutic inference. Integrated phenotypic and genomic surveillance is therefore required to support evidence-based antimicrobial stewardship in canine pseudomonal otitis.

## Simple Summary

Chronic canine otitis externa caused by *Pseudomonas aeruginosa* is difficult to manage because the bacterium combines intrinsic resistance with rapid adaptive responses. We examined an archival Hungarian collection obtained in 2010 and 2017. Minimum inhibitory concentrations (MICs) were determined for 13 antimicrobial agents and chlorhexidine in 67 isolates, and 59 isolates had matched whole-genome data. Fluoroquinolone non-susceptibility was frequent under current canine-specific interpretive criteria, whereas aminoglycoside and ceftazidime MICs were generally low. Piperacillin–tazobactam displayed a broad, frequently non-wild-type distribution. The genomic background was dominated by conserved efflux systems, intrinsic β-lactamases, and aminoglycoside-, peptide-, and fluoroquinolone-associated determinants. The ciprofloxacin-modifying determinant *crpP* occurred in a subset of isolates, including six isolates in which *crpP* occurred on contigs predicted by PlasFlow to be plasmid-like, but neither individual determinants nor total resistance-gene burden reliably predicted MICs after correction for multiple testing. These findings show that genomic detection of resistance-associated genes cannot replace standardized susceptibility testing and reinforce the need for culture- and MIC-guided therapy in canine pseudomonal otitis.

## 1. Introduction

Otitis externa is among the most frequent disorders encountered in canine primary care, and its occurrence is shaped by breed, ear conformation, allergic disease, moisture exposure, and chronic structural change of the external ear canal [1,2,3]. Bacterial proliferation is usually secondary to an altered local environment rather than the primary cause of inflammation. Nevertheless, once *Pseudomonas aeruginosa* becomes established, the disease often evolves into a painful, suppurative, and recurrent syndrome that is difficult to eradicate [2,3,4]. Recent surveys from Europe and Asia continue to identify *P. aeruginosa* as one of the most clinically consequential organisms in chronic or recurrent canine otitis, with substantial geographic variation in antimicrobial susceptibility [3,4,5,6,7,8,9,10,11].

The therapeutic difficulty of pseudomonal otitis reflects the biology of the organism. *P. aeruginosa* combines a poorly permeable outer membrane, inducible chromosomal β-lactamase activity, multiple resistance–nodulation–division efflux systems, target-site mutability, and a pronounced capacity for biofilm formation [12,13,14,15,16]. These mechanisms interact rather than operate independently. A genome can therefore contain an extensive intrinsic resistance repertoire without expressing a high minimum inhibitory concentration (MIC) against antimicrobials under standardized planktonic in vitro conditions, while regulatory mutations, gene dosage, promoter activity, growth state, or biofilm physiology may produce phenotypic resistance that is not explained by simple gene-presence screening [17].

This distinction is particularly important in veterinary otology. Topical products may achieve concentrations that exceed systemic exposure, but local activity is modified by exudate, biofilm, canal architecture, contact time, and ototoxicity constraints. Conversely, systemic therapy may be required when the middle ear or deeper tissues are involved. Standardized MIC testing remains the most reproducible laboratory measure for susceptibility surveillance, but its clinical interpretation requires organism-, host- and drug-specific breakpoints or, where these are unavailable, epidemiological cut-off values that distinguish wild-type from non-wild-type populations without claiming clinical efficacy [17,18,19,20].

Companion-animal *P. aeruginosa* also has One Health relevance. The species is ubiquitous in water, soil, domestic environments, and clinical settings, and genetically diverse lineages can circulate across ecological compartments [21,22,23,24]. Recent Hungarian veterinary studies have similarly emphasized that antimicrobial resistance must be monitored across both managed and free-living animal reservoirs, because the occurrence and dissemination of resistant organisms are strongly influenced by host population, production system and ecological setting [25,26]. Recent genomic studies of canine otitis isolates have shown broad population diversity, occasional recovery of sequence types associated with human high-risk clones, conserved intrinsic resistance repertoires, and imperfect agreement between genotype and phenotype [4,5,7,8,9,10]. Beyond companion animals, recent veterinary studies have also demonstrated the complementary value of resistome profiling, phenotypic susceptibility testing, and mobile-genetic-element analysis when characterizing antimicrobial resistance in animal-derived bacterial populations. The public-health implication is not that canine otitis is uniformly zoonotic but that resistant companion-animal isolates belong within integrated surveillance systems because they can reveal shared resistance mechanisms and mobile genetic contexts [27,28].

The present study investigates a historical Hungarian collection of canine otitis externa-associated *P. aeruginosa* obtained in 2010 and 2017. Our objectives were to (i) define MIC distributions across a broad antipseudomonal panel and chlorhexidine; (ii) compare the 2010 and 2017 subsets; (iii) characterize resistance-associated genes and their predicted mobile-genetic-element proximity and plasmid-like contig context; and (iv) test whether individual genomic features or overall resistance-gene burden explained the observed MIC variation. A limited panel of virulence-associated loci was retained only to provide contextual information in the integrated genomic visualizations. We hypothesized that the historical collection would contain a conserved intrinsic resistance backbone, but that gene presence alone would incompletely predict quantitative susceptibility.

## 2. Materials and Methods

### 2.1. Study Design and Bacterial Isolates

This study used an archival strain collection maintained at the Department of Pharmacology and Toxicology, University of Veterinary Medicine Budapest. Isolates originated from ear swabs collected from dogs diagnosed with otitis externa during routine veterinary care in Hungary in 2010 or 2017. Only one clinical isolate per dog was included in this study, and no dog contributed more than one isolate to the analytical dataset. Primary isolation was performed in a diagnostic laboratory using selective cetrimide medium (Biolab Zrt., Budapest, Hungary), colony morphology, and characteristic pigment production. Species-level identification was subsequently confirmed by matrix-assisted laser desorption/ionization time-of-flight mass spectrometry (MALDI-TOF MS) using a Microflex LT instrument and the MALDI Biotyper system (Bruker Daltonics, Bremen, Germany) equipped with MBT Compass software v4.1 and the 2026 reference library. Only isolates identified as *P. aeruginosa* according to the manufacturer’s acceptance criteria were included. Isolates were cryopreserved in Microbank systems (VWR International Kft., Debrecen, Hungary) at −80 °C until analysis. No prospective sampling, intervention, or experimental procedure involving live animals was performed for the present study.

The identifier range assigned to the historical collection comprised nominal isolate numbers 70–139. The curated analytical dataset comprised complete MIC measurements for 67 clinical isolates, including 32 isolates collected in 2010 and 35 collected in 2017. Matched whole-genome results were available for 59 of these isolates. Analyses were therefore conducted on the complete phenotypic dataset (*n* = 67) and on the matched phenotype–genotype subset (*n* = 59), as appropriate.

### 2.2. Broth Microdilution Susceptibility Testing

MICs were determined by broth microdilution in accordance with Clinical and Laboratory Standards Institute (CLSI) principles [17,29]. Frozen isolates were recovered in cation-adjusted Mueller–Hinton broth (CAMHB) and incubated aerobically at 37 °C overnight. Antimicrobial stock solutions were prepared from analytical-grade powders with potency correction and sterile filtration. Twofold dilution series were dispensed into 96-well microtiter plates over ranges selected to encompass the expected MIC distributions. Colonies were suspended in sterile saline to a turbidity equivalent to a 0.5 McFarland standard and diluted in CAMHB to yield a final inoculum of approximately 5 × 10^5^ colony-forming units (CFU)/mL in each well. Positive-growth and sterility controls were included. *Pseudomonas aeruginosa* ATCC 27853 was used as the quality-control strain, and each run was accepted only when the corresponding MIC values were within the CLSI-recommended ranges. Plates were incubated aerobically at 37 °C for 18–24 h and then read visually; the MIC was the lowest concentration preventing visible growth. Each isolate–compound combination was tested in duplicate, and discordant duplicate endpoints were repeated.

The panel comprised enrofloxacin, marbofloxacin, ciprofloxacin, norfloxacin, orbifloxacin, tobramycin, gentamicin, amikacin, ceftazidime, cefoperazone, imipenem, polymyxin B, piperacillin–tazobactam, and chlorhexidine. For piperacillin–tazobactam, piperacillin was tested in twofold serial dilutions, while tazobactam was maintained at a fixed concentration of 4 µg/mL. Concentrations are reported in µg/mL. Chlorhexidine was included as a topical antiseptic and interpreted descriptively because no validated clinical breakpoint or epidemiological cut-off is available for canine otitis-associated *P. aeruginosa*.

### 2.3. Interpretive Criteria

Enrofloxacin and marbofloxacin were categorized using canine-specific *P. aeruginosa* MIC breakpoints from CLSI VET01S, 7th edition (2024), Table 2B [18,19]. Enrofloxacin was interpreted as susceptible at ≤0.06 µg/mL, susceptible dose-dependent at 0.12–0.25 µg/mL, and resistant at ≥0.5 µg/mL; marbofloxacin was interpreted as susceptible at ≤0.12 µg/mL, susceptible dose-dependent at 0.25 µg/mL, and resistant at ≥0.5 µg/mL. In the experimental twofold-dilution series, the tested concentration of 0.125 µg/mL corresponds to the CLSI-reported 0.12 µg/mL dilution step and was categorized accordingly. European Committee on Antimicrobial Susceptibility Testing (EUCAST) epidemiological cut-off values (ECOFFs) for *P. aeruginosa* were obtained from the species-specific MIC distributions in the EUCAST MIC and Zone Diameter Distribution database (accessed 12 July 2026) [20]. The ECOFFs applied were 0.5 µg/mL for ciprofloxacin, 2 µg/mL for tobramycin, 8 µg/mL for gentamicin, 16 µg/mL for amikacin, 8 µg/mL for ceftazidime, 4 µg/mL for imipenem, and 16 µg/mL for piperacillin–tazobactam. ECOFFs distinguish wild-type from non-wild-type populations and were not interpreted as veterinary clinical breakpoints. Cefoperazone, norfloxacin, orbifloxacin, polymyxin B, and chlorhexidine were summarized descriptively because validated canine *P. aeruginosa* criteria were unavailable or not directly transferable.

### 2.4. DNA Extraction, Nanopore Sequencing, and Assembly

Genomic DNA was extracted using the Quick-DNA Fungal/Bacterial Miniprep Kit (Zymo Research, Irvine, CA, USA) and quantified fluorometrically. Libraries were prepared with the Oxford Nanopore Technologies Native Barcoding Kit 24 V14 (SQK-NBD114.24, Oxford Nanopore Technologies, Oxford, UK) and sequenced on R10.4.1 MinION flow cells using a MinION Mk1B device. Basecalling and demultiplexing were performed with Dorado v7.6.8 through MinKNOW v24.11.10 in super-accuracy mode. Read preprocessing used NanoLyse v1.2.1 and NanoFilt v2.8.0; 50 bases were trimmed from each read end, and reads with a mean quality score < 8 or length < 500 bp were removed. Read-quality summaries were generated with NanoPlot as implemented in NanoPack [30].

Long-read de novo assembly was performed with Flye v2.9.5-b1801 [31]. Assemblies were polished iteratively by mapping reads with minimap2 v2.28 [32], correcting with Racon v1.5.0 [33], and applying Medaka v2.0.1 with the model appropriate for the R10.4.1 super-accuracy data. Assembly statistics were generated using QUAST v5.0.2 [34]. The whole-genome assemblies were submitted to National Center for Biotechnology Information (NCBI) GenBank under BioProject accession PRJNA1499158 and BioSample accessions SAMN61867835–SAMN61867893.

### 2.5. Genome Quality Assessment

Genome completeness was evaluated with BUSCO v5.8.2 in auto-lineage mode [35], and completeness, contamination, and strain heterogeneity were estimated with CheckM v1.2.4 [36]. Assemblies with CheckM completeness ≥ 95% and contamination ≤ 5% were considered high quality for absence-sensitive interpretation. Assemblies outside these thresholds were retained for transparent presence-based descriptive screening because a high-confidence positive gene call is less vulnerable to assembly incompleteness than a negative call; their absence profiles were interpreted cautiously.

### 2.6. Resistance-Determinant, Mobile-Element, and Contextual Marker Screening

Resistance-associated determinants were identified with ABRicate v1.4.0 against the Comprehensive Antibiotic Resistance Database (CARD; v4.0.1) [37,38]. Primary presence calls required both nucleotide identity and reference coverage of at least 80%. Genes were summarized at the individual-gene and functional-system levels. Because *P. aeruginosa* resistance often depends on regulatory mutations and expression rather than simple gene presence, CARD hits were treated as resistance-associated genomic features and not as proof of phenotypic resistance. The genomic analysis was intentionally restricted to determinant-presence screening and did not include systematic variant calling of quinolone-resistance-determining regions (QRDRs) in *gyrA*, *gyrB*, *parC*, or *parE*.

Mobile genetic elements were detected with MobileElementFinder v1.0.5 [39]. For each resistance-gene locus, the distance to a predicted mobile element was calculated and recorded at nested thresholds of ≤1 kb, ≤10 kb, and ≤50 kb. Counts at these thresholds were not summed because every locus meeting a shorter threshold also meets the longer thresholds. Contigs were classified as chromosome- or plasmid-like with PlasFlow v1.1 [40]. Plasmid assignment was considered predictive rather than definitive in the absence of circularization and replicon confirmation. The analytical strategy followed previously described distance-aware reporting principles [41].

To provide limited pathogenic context for the integrated genomic visualizations, a prespecified panel of virulence-associated loci (*exoS*, *exoU*, *toxA*, *lasB*, *algD*, *pvdS*, and *hcp1*) was annotated using ABRicate v1.4.0 against the Virulence Factor Database (VFDB; accessed 2 April 2026) using the same ≥80% nucleotide-identity and ≥80% reference-coverage thresholds [42]. These markers were reported descriptively and were not used to infer comprehensive virulome architecture.

### 2.7. Statistical Analysis

**Figure 1:**
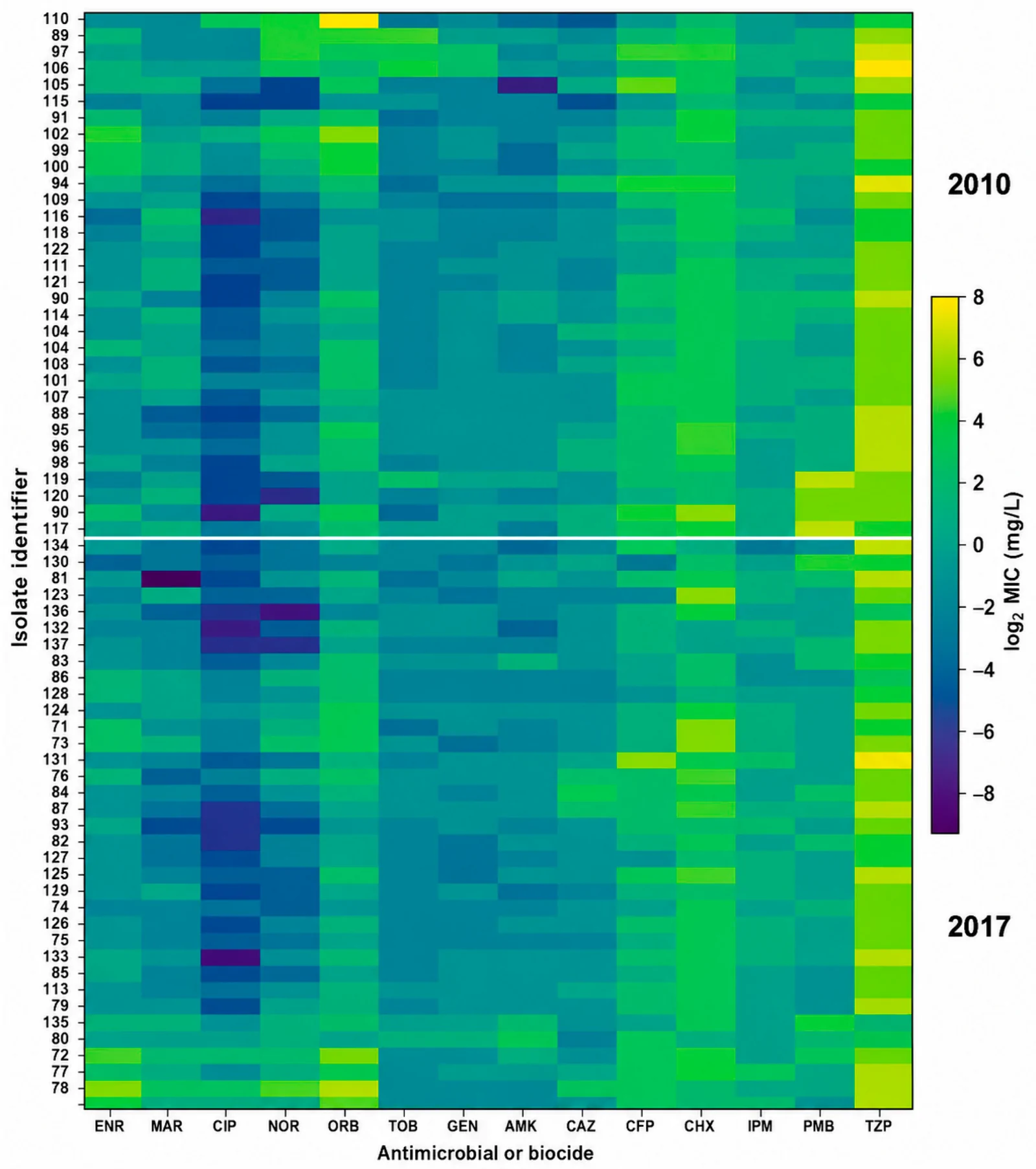
Quantitative minimum inhibitory concentration (MIC) landscape of the 67 archival isolates. Values are displayed on a log_2_ scale. MIC profiles were standardized by antimicrobial agent across the complete dataset, and isolates were hierarchically clustered separately within each sampling year using Euclidean distance and average linkage. ENR, enrofloxacin; MAR, marbofloxacin; CIP, ciprofloxacin; NOR, norfloxacin; ORB, orbifloxacin; TOB, tobramycin; GEN, gentamicin; AMK, amikacin; CAZ, ceftazidime; CFP, cefoperazone; CHX, chlorhexidine; IPM, imipenem; PMB, polymyxin B; TZP, piperacillin–tazobactam.

MIC values were transformed to the log_2_ scale for inferential analyses. For Figure 1, log_2_-transformed MIC values were standardized by antimicrobial agent across the complete dataset, and isolates were hierarchically clustered separately within each sampling year using Euclidean distance and average linkage. MIC_50_ and MIC_90_ were defined as the lowest tested concentrations inhibiting at least 50% and 90% of isolates, respectively. Continuous MIC distributions from 2010 and 2017 were compared with two-sided Mann–Whitney U tests. Differences in resistant or non-wild-type proportions were tested with Fisher’s exact test. For matched genomes, MIC distributions were compared between determinant-positive and determinant-negative isolates when both groups contained sufficient observations. Spearman rank correlation tested associations between the number of resistance-associated CARD calls and log_2_ MIC. Benjamini–Hochberg correction was applied within each family of comparisons to control the false discovery rate (FDR), and an adjusted q value < 0.05 was considered significant. Sample Python (version 3.13) code implementing the principal statistical, phenotype–genotype, sensitivity, and mobile-element-context analyses is publicly available in a dedicated GitHub repository: https://github.com/kerekadam03/canine-otitis-pseudomonas-analysis (accessed on 18 August 2026).

As a sensitivity analysis, determinant prevalence, prespecified determinant–MIC comparisons, and correlations between total CARD-call burden and MIC were repeated after exclusion of the six assemblies that did not meet the predefined CheckM quality criteria.

## 3. Results

### 3.1. Cohort Composition and Genome Quality

The phenotypic dataset comprised 67 isolates, including 32 collected in 2010 and 35 collected in 2017. All isolates included in the phenotypic dataset were confirmed as *P. aeruginosa* by MALDI-TOF MS. Matched genomic results were available for 59 isolates. CheckM completeness had a median of 99.68% and ranged from 88.29% to 100%, while median contamination was 0.14%. Fifty-three assemblies met the prespecified high-quality threshold. Six assemblies (identifiers 77, 110, 119, 120, 126, and 137) were flagged because of completeness < 95%, contamination > 5%, or both. The two assemblies with very high CheckM contamination estimates were treated as potentially mixed or duplicated, and negative gene calls from these assemblies were interpreted with particular caution. Complete isolate-level MIC data, sequencing availability, genome-quality metrics, and selected genomic markers are provided in Table S1.

### 3.2. MIC Distributions

MIC distributions were heterogeneous across isolates and compounds (Figure 1; Table 1). Fluoroquinolone MICs spanned wide ranges: enrofloxacin 0.06–32 µg/mL, marbofloxacin 0.001–8 µg/mL, ciprofloxacin 0.004–8 µg/mL, norfloxacin 0.004–32 µg/mL, and orbifloxacin 0.25–256 µg/mL. Enrofloxacin and marbofloxacin MIC_50_ values were both 0.5 µg/mL, with MIC_90_ values of 4 and 2 µg/mL, respectively. Aminoglycoside distributions were generally lower: tobramycin, gentamicin, and amikacin MIC_50_ values were 0.25, 0.5, and 0.5 µg/mL, respectively. Ceftazidime and imipenem MIC_90_ values were 2 and 4 µg/mL. Piperacillin–tazobactam showed the highest central distribution among the β-lactams tested (MIC_50_ 32 µg/mL; MIC_90_ 64 µg/mL). Chlorhexidine MICs ranged from 1 to 32 µg/mL, with MIC_50_ and MIC_90_ values of 8 and 16 µg/mL.

### 3.3. Interpretation Against Available Breakpoints and ECOFFs

Under current canine-specific CLSI breakpoints, 59/67 (88.1%) isolates were resistant to enrofloxacin, 7 (10.4%) were susceptible dose-dependent, and 1 (1.5%) was susceptible. For marbofloxacin, 43/67 (64.2%) were resistant, 19 (28.4%) were susceptible dose-dependent, and 5 (7.5%) were susceptible. EUCAST epidemiological cut-off (ECOFF)-based analysis classified 7 isolates (10.4%) as ciprofloxacin non-wild-type, 4 (6.0%) as tobramycin non-wild-type, 1 (1.5%) as imipenem non-wild-type, and 51 (76.1%) as piperacillin–tazobactam non-wild-type. All isolates remained within the available wild-type distributions for ceftazidime, gentamicin, and amikacin (Table 2). These ecological classifications should not be interpreted as direct predictions of clinical response in canine ears.

### 3.4. Temporal Comparison of the 2010 and 2017 Subsets

**Figure 2:**
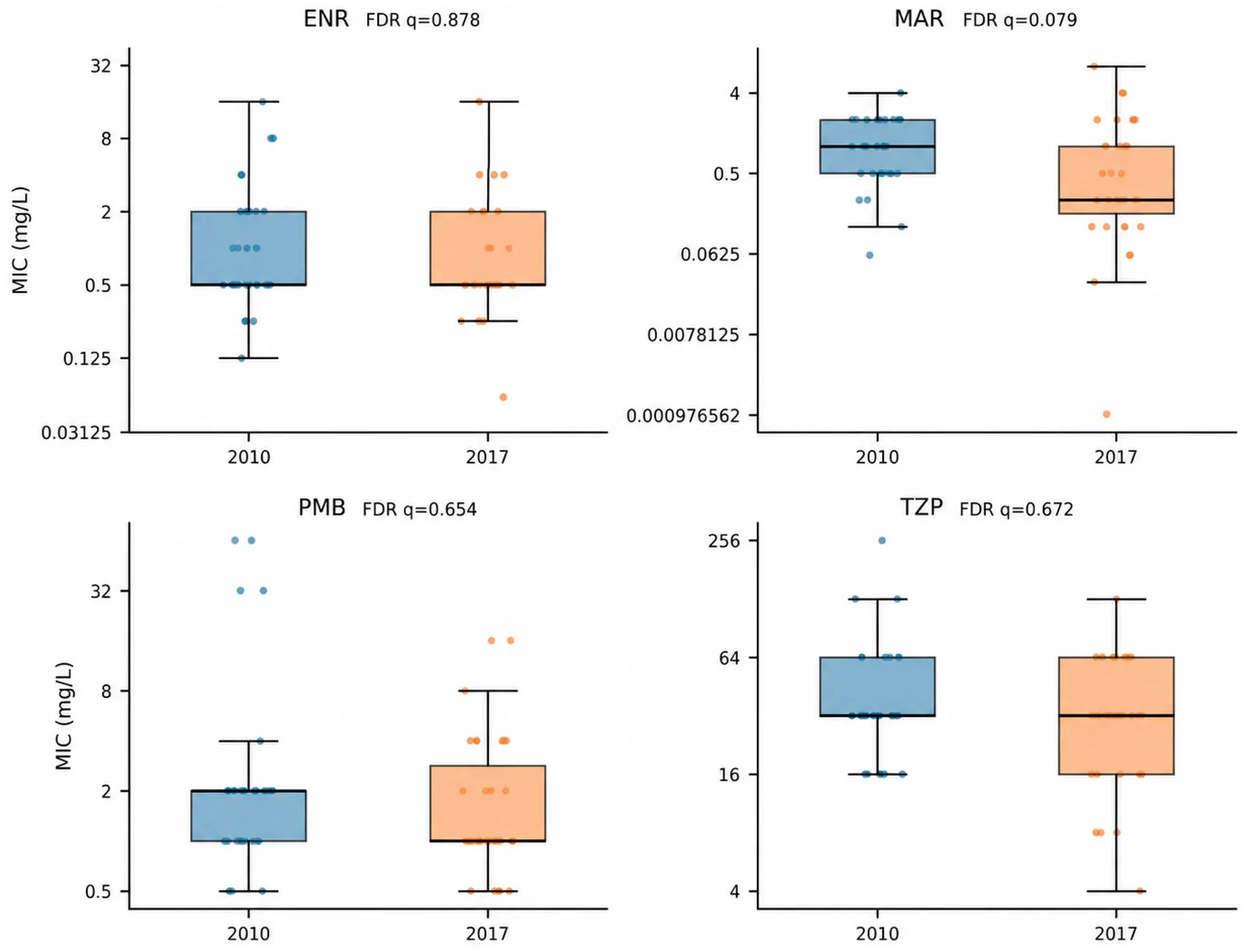
Temporal comparison of selected minimum inhibitory concentration (MIC) distributions. Boxes show the interquartile range and median; whiskers indicate the non-outlier range, and points represent individual isolates. q values are Benjamini–Hochberg-adjusted results from Mann–Whitney U tests performed on log_2_-transformed MICs. ENR, enrofloxacin; MAR, marbofloxacin; PMB, polymyxin B; TZP, piperacillin–tazobactam.

After correction across the 14 continuous MIC comparisons, no compound showed a significant temporal shift (all q ≥ 0.079; Figure 2). Marbofloxacin displayed the largest nominal change: the median MIC decreased from 1 µg/mL in 2010 to 0.25 µg/mL in 2017 (unadjusted *p* = 0.0057; q = 0.079). When the same data were categorized using the current canine breakpoint, the resistant proportion decreased from 28/32 (87.5%) in 2010 to 15/35 (42.9%) in 2017 (Fisher’s exact *p* = 0.00026; q = 0.0024; odds ratio for resistance in 2017 versus 2010, 0.11; 95% confidence interval (CI), 0.03–0.37). Enrofloxacin resistance remained essentially unchanged (87.5% versus 88.6%). The four tobramycin non-wild-type isolates all originated from 2010, but this difference did not remain significant after correction (q = 0.211).

### 3.5. Resistance-Associated Genomic Repertoire

CARD screening of the 59 matched genomes identified 64 unique resistance-associated gene calls meeting the 80% identity and coverage thresholds. The genomic background was dominated by conserved intrinsic systems. Complete or near-complete components of MexAB–OprM, MexCD–OprJ, MexEF–OprN, MexXY–OprM, MuxABC–OpmB, TriABC–OpmH, MexGHI–OpmD, MexPQ–OpmE, and MexVW–OprM were detected in 57–58 isolates. *APH(3′)-IIb*, *arnA*, *fosA*, PDC β-lactamase alleles, and OXA-50-like β-lactamase alleles each occurred in 58/59 genomes, while *basS* occurred in 57/59 (Table 3). These near-universal determinants defined a broad intrinsic resistance backbone but provided little discrimination among isolates with different MICs.

**Figure 3:**
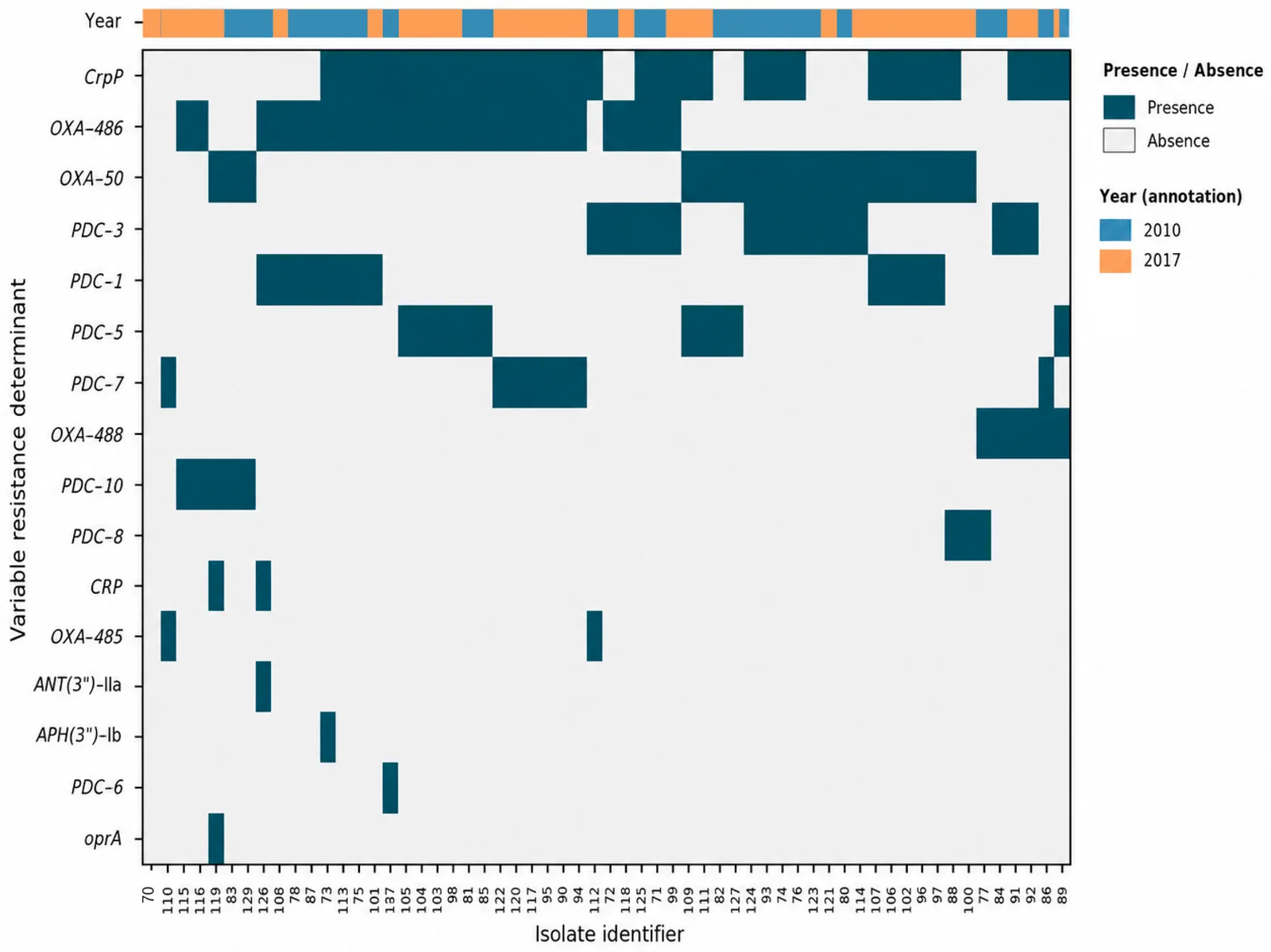
Distribution of variable resistance-associated determinants across the 59 matched genomes. The upper annotation band indicates isolates collected in 2010 (blue) and 2017 (orange). Dark teal and light grey cells denote determinant presence and absence, respectively, at ≥80% nucleotide identity and ≥80% reference coverage. Ubiquitous efflux-system components were omitted to preserve the resolution of variable features.

The main variable determinants were *crpP* and allele-level β-lactamase calls (Figure 3). *crpP* was present in 37/59 genomes. PDC-3 (17/59), PDC-1 (13/59), PDC-5 (11/59), PDC-7 (8/59), PDC-10 (5/59), PDC-8 (3/59), and PDC-6 (1/59) represented the most frequent PDC variants. OXA-486 (28/59), OXA-50 (22/59), OXA-488 (6/59), and OXA-485 (2/59) accounted for the OXA-50-like family. Rare, acquired aminoglycoside-associated calls included *ANT(3″)-IIa* and *APH(3″)-Ib* in one isolate each.

### 3.6. Predicted Mobile-Genetic-Element Context

Among the clinically relevant resistance-associated genes, 138 unique loci were located within 50 kb of at least one predicted mobile genetic element; 18 of these were within 10 kb, and 1 was within 1 kb. At the association level, 166 unique resistance-gene–mobile-element pairs occurred within 50 kb. Because the thresholds were nested, these values describe progressively stricter subsets rather than additive totals. PlasFlow classified seven *crpP*-bearing contigs from six isolates (73, 91, 94, 101, 117, and 120) as plasmid-like; isolate 73 contained two such loci. Contig lengths, *crpP* coordinates, circularization status, and predicted mobile-element proximity are provided in Table S5. Circularization was not confirmed for any of these contigs. Only the *crpP*-bearing contig of isolate 120 contained predicted mobile elements within 50 kb, comprising two IS222 predictions at distances of 22.5 and 36.9 kb from the *crpP* locus; no plasmid-predicted *crpP* locus was located within 10 kb of a predicted mobile element. These assignments remain computational predictions and do not establish autonomous plasmid structure or transferability.

### 3.7. Phenotype–Genotype Relationships

**Figure 4:**
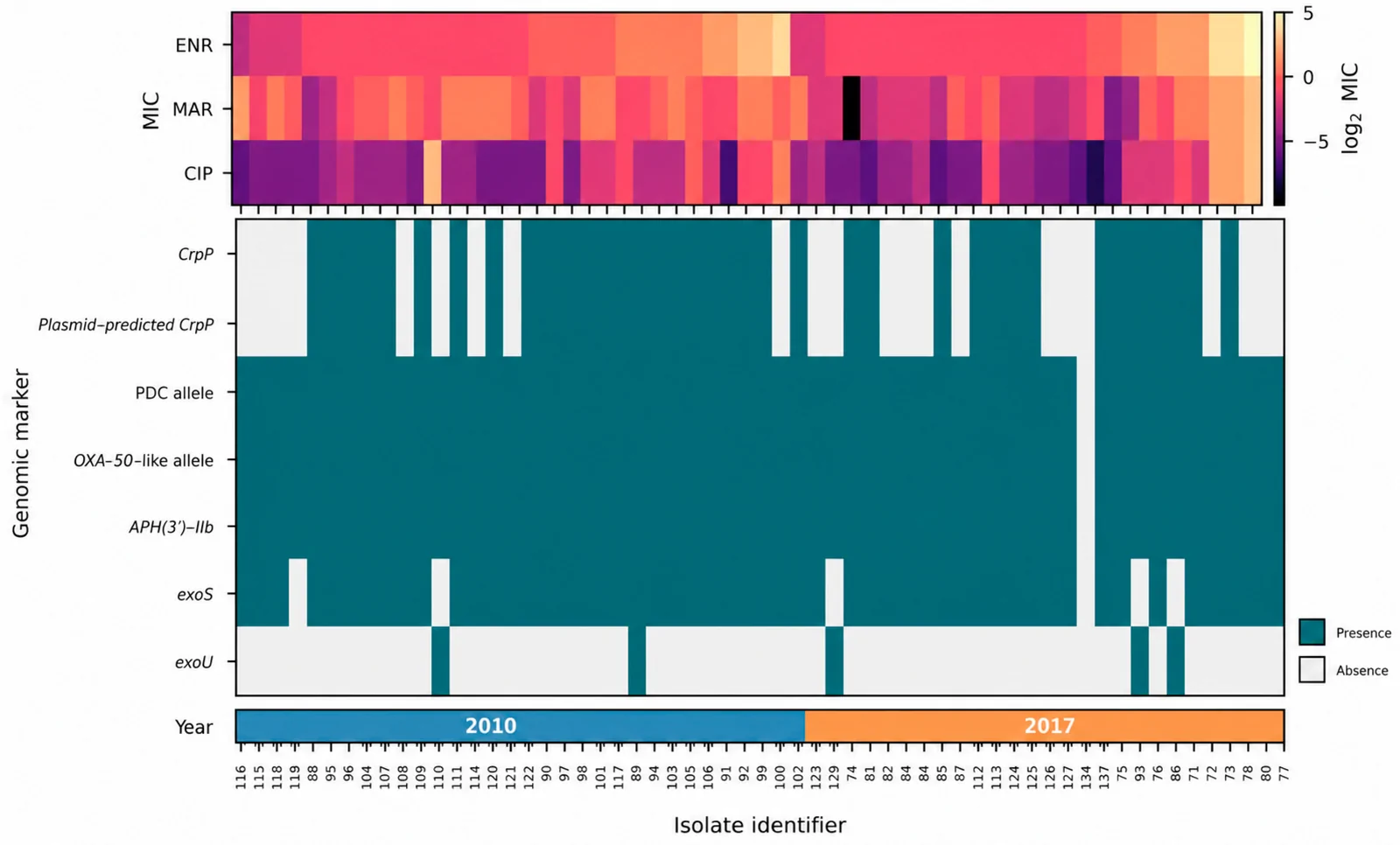
Integrated phenotype–genotype profile of the 59 matched isolates. Quantitative fluoroquinolone minimum inhibitory concentrations are shown on a log_2_ scale in the upper panel. The lower panel shows binary resistance-associated markers together with two contextual type III secretion effector markers, *exoS* and *exoU*. Teal and light grey denote marker presence and absence, respectively. Isolates are ordered by sampling year and enrofloxacin MIC; the lower annotation band indicates 2010 (blue) and 2017 (orange).

No tested determinant–MIC association remained significant after false-discovery-rate correction (all q ≥ 0.291; Table S3). *crpP*-positive isolates had a higher enrofloxacin MIC50 than *crpP*-negative isolates (1 versus 0.5 µg/mL), but the nominal association (*p* = 0.025) was not retained after correction (q = 0.291). Plasmid-predicted *crpP* similarly showed no significant association with any fluoroquinolone MIC. *APH(3′)-IIb* presence did not explain tobramycin, gentamicin, or amikacin MIC variation, and *arnA* or *basS* presence did not significantly distinguish polymyxin B MICs (Figure 4). The total number of CARD calls per genome was not significantly correlated with any MIC after correction (all q ≥ 0.521; Table S2).

A sensitivity analysis restricted to the 53 high-quality assemblies produced the same overall interpretation. Key determinant prevalences remained similar (*crpP*, 35/53; *APH(3′)-IIb*, 52/53; *arnA*, 52/53; *basS*, 52/53; plasmid-predicted *crpP*, 5/53). No prespecified determinant–MIC association remained significant after FDR correction (all q ≥ 0.209), and no correlation between total CARD-call burden and MIC remained significant (all q ≥ 0.118; Table S4).

### 3.8. Selected Contextual Virulence Markers

**Figure 5:**
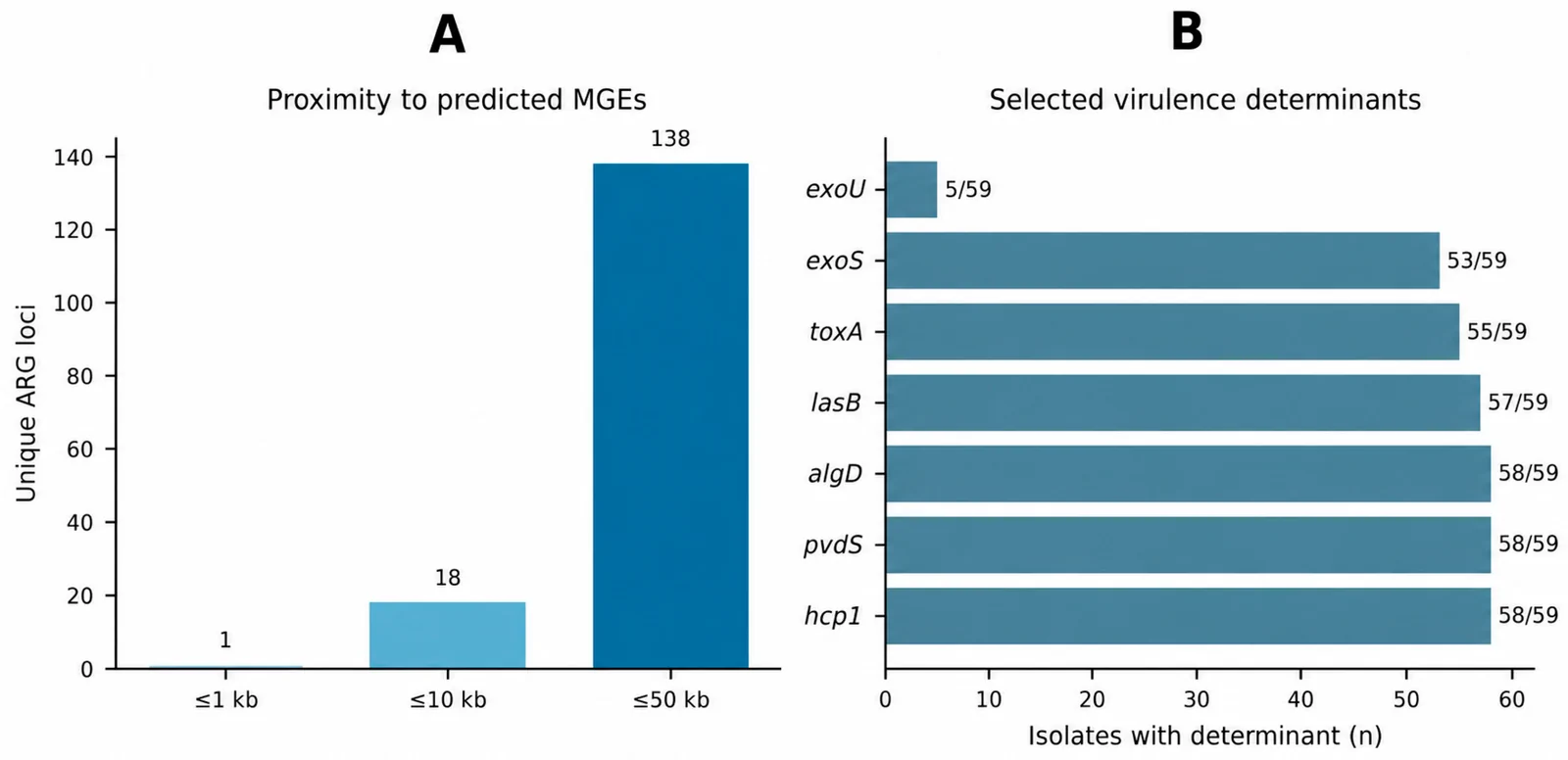
Mobile-genetic-element proximity and selected contextual virulence-associated determinants. (**A**) Number of unique resistance-gene loci located within nested distances of predicted mobile genetic elements. Because the distance thresholds are nested, the displayed counts represent progressively broader subsets and are not additive. (**B**) Prevalence of the prespecified contextual VFDB markers among the 59 matched genomes; labels at the ends of the bars indicate the number of positive genomes divided by the total number examined. MGE, mobile genetic element; ARG, antimicrobial resistance gene.

Selected virulence-associated loci were retained solely to provide pathogenic context for the resistance-focused genomic analysis (Figure 5). The type III secretion effector genes *exoS* and *exoU* were detected in 53/59 and 5/59 genomes, respectively. The selected contextual markers *toxA*, *lasB*, *algD*, *pvdS*, and *hcp1* occurred in 55/59, 57/59, 58/59, 58/59, and 58/59 genomes, respectively.

## 4. Discussion

This study links quantitative MIC data with long-read genome screening in a historical collection of canine otitis-associated *P. aeruginosa*. Fluoroquinolone non-susceptibility was frequent under current canine-specific breakpoints, whereas aminoglycoside and ceftazidime MICs were generally low. The MIC distributions from 2010 and 2017 were broadly stable after correction for multiple testing, although categorical marbofloxacin resistance was lower in 2017. The resistance-associated genomic repertoire was dominated by a conserved intrinsic backbone, but MICs varied by several orders of magnitude, and neither individual gene-presence calls nor total resistance-gene burden adequately explained this variation. Genomic screening is therefore informative for surveillance and mechanistic interpretation but cannot replace standardized phenotyping for therapeutic decisions.

The enrofloxacin-resistant proportion of 88.1% is high compared with the 25.4% reported in a recent multinational European collection using disk diffusion [8], and it exceeds several recent regional estimates [5,6,10]. Direct comparison is nevertheless constrained by study period, sampling frame, method, and interpretive criteria. The current canine CLSI breakpoints are substantially lower than many historical thresholds and were designed around canine pharmacokinetic–pharmacodynamic targets. Their application can therefore reclassify isolates that would previously have been reported as susceptible or intermediate. This is not a technical artifact but a consequence of more conservative, dose-aware interpretation [18,29].

The divergence between enrofloxacin and marbofloxacin is also instructive. Both had an overall MIC_50_ of 0.5 µg/mL, yet the breakpoint structures and year-specific distributions produced different categorical patterns. Marbofloxacin resistance declined markedly in 2017, whereas enrofloxacin resistance remained stable. The result should not be interpreted as proof of a population-wide temporal trend because the isolates were archival convenience samples, the two years were not continuously sampled, and treatment histories were unavailable. Nonetheless, the magnitude and adjusted significance justify reporting and show why raw MIC distributions should accompany categorical summaries.

Recent studies have likewise documented heterogeneous fluoroquinolone resistance in canine *P. aeruginosa*. Polish isolates showed frequent resistance-associated phenotypes and exotoxin genes [6], Romanian isolates remained uniformly susceptible to aminoglycosides but showed substantial fluoroquinolone resistance [10], and a large European dataset identified fluoroquinolone resistance as the dominant phenotypic concern [8]. A later, independent Hungarian 2024 cohort showed even more extensive elevation of MICs under the same current interpretive logic [43]. Together, these studies suggest that regional surveillance must be time-resolved and methodology-aware rather than relying on imported prevalence estimates. This principle extends beyond *P. aeruginosa*: broth-microdilution surveillance of *Pasteurella multocida* from different host species and geographic origins has likewise demonstrated substantial population-dependent variation in antimicrobial susceptibility, underscoring the limitations of extrapolating susceptibility patterns between unrelated veterinary populations [44].

The low gentamicin, tobramycin, and amikacin MICs in most historical isolates are consistent with several recent canine otitis datasets [8,10]. The four tobramycin non-wild-type isolates were confined to 2010, but the year effect did not survive correction. Near-universal *APH(3′)-IIb* and MexXY–OprM components did not correspond to uniformly high aminoglycoside MICs, emphasizing that intrinsic gene presence does not imply constitutive high-level expression. Aminoglycoside resistance can additionally involve regulatory changes, permeability, ribosomal targets, and acquired modifying enzymes, none of which is fully captured by a binary gene matrix [12,13].

Ceftazidime MICs remained within the EUCAST wild-type distribution, and only one isolate was imipenem non-wild type. This differs from settings in which carbapenem-resistant veterinary isolates and high-risk clones have been recovered [7,45]. The absence of a strong carbapenem phenotype is reassuring but should not be overgeneralized beyond the sampled period. By contrast, piperacillin–tazobactam MICs were frequently above the adopted ECOFF. Chromosomal PDC and OXA-50-like β-lactamases were nearly universal, but allele presence alone did not explain the broad distribution. AmpC expression, efflux, permeability, and target changes can act synergistically, and sequence-based interpretation requires regulatory and mutational analysis beyond cataloguing β-lactamase genes [12,15]. The high proportion of piperacillin–tazobactam non-wild-type isolates reflects an ECOFF-based population classification and should not be interpreted as a corresponding rate of clinical resistance or treatment failure in canine otitis.

Polymyxin B MICs were mostly low but included a long upper tail. *arnA* and *basS* were almost universal and therefore had little discriminatory value. Because validated canine otitis breakpoints were unavailable and polymyxin B is commonly administered topically, this study deliberately avoided assigning clinical susceptibility categories. The same principle applies to chlorhexidine. Its MIC_50_ of 8 µg/mL and MIC_90_ of 16 µg/mL provide a comparative baseline for the collection, but antiseptic efficacy in vivo depends on formulation, concentration, organic burden, contact time, and tissue tolerability. Recent veterinary literature on topical and non-antibiotic approaches—including essential oils, bacteriophages, and stabilized hypochlorous acid—illustrates the continuing search for non-systemic strategies against difficult-to-treat bacterial infections. Stabilized hypochlorous acid has documented broad-spectrum bactericidal activity, including activity against *P. aeruginosa*, although formulation-specific efficacy, otic tolerability, and clinical performance require direct validation [46,47,48].

The genomic results closely parallel recent canine whole-genome sequencing (WGS) studies in showing a remarkably conserved intrinsic resistance-gene complement despite marked phenotypic heterogeneity [8,9,43]. Efflux components, PDC, OXA-50-like β-lactamases, *fosA*, *arnA*, and *APH(3′)-IIb* were present in almost every genome. These genes are biologically important, but their near ubiquity limits their value as isolate-level biomarkers. In *P. aeruginosa*, resistance frequently emerges through altered expression or point mutations in regulatory and target genes. A presence/absence pipeline based on assembled sequence will miss promoter architecture, expression state, gene amplification, heteroresistance, and adaptive biofilm phenotypes [12,13,14,15,16].

The absence of significant associations after multiplicity correction indicates that gene-presence data had limited predictive value for MIC variation in this collection. The nominal association between *crpP* and enrofloxacin MIC is mechanistically plausible because CrpP was first described as a plasmid-encoded ciprofloxacin-modifying enzyme [49]. However, the effect was modest and inconsistent across fluoroquinolones, and only six isolates carried *crpP* on contigs classified as plasmid-like. *crpP* variants can differ in activity. Importantly, the present gene-presence framework did not systematically interrogate QRDR substitutions in *gyrA*, *gyrB*, *parC*, or *parE* or regulatory variants affecting efflux. These mechanisms are major contributors to fluoroquinolone resistance in *P. aeruginosa* and may account for part of the phenotype–genotype discordance observed here. Targeted variant calling together with efflux-regulatory analysis should therefore be incorporated into future work. Accordingly, *crpP* should be regarded as a contextual marker rather than a standalone predictor.

A substantial number of resistance-gene loci occurred within 50 kb of predicted mobile elements, but only one lay within 1 kb. This distance gradient is more informative than a single broad proximity cut-off. Close physical linkage increases plausibility of co-mobilization, whereas a 50 kb association may simply place two features in the same genomic neighborhood. Likewise, PlasFlow classification provides probabilistic evidence and cannot by itself demonstrate an autonomous transferable plasmid. Long-read assemblies create an opportunity for stronger locus reconstruction, but targeted circularization, replicon analysis, conjugation, or transformation experiments are required before horizontal-transfer claims are justified [39,40,41].

The One Health relevance of the collection arises from the combination of a broad intrinsic resistance-gene repertoire, occasional mobile-associated determinants, and an ecological organism capable of occupying animal, human, and environmental reservoirs [22,23,24,50]. It does not follow that these isolates were transmitted between dogs and humans. Recent canine genomic studies have instead shown a predominantly diverse population punctuated by occasional sequence types linked to human high-risk clones [7,8,9,51,52]. Because lineage-level population structure was outside the scope of the present resistance-focused analysis, no lineage-level transmission inferences were made. Genomic similarity and mobile-element context should be quantified directly rather than inferred from host origin alone.

Selected virulence-associated markers were retained only to provide pathogenic context for the resistance-focused analysis. The predominance of *exoS* and minority occurrence of *exoU* indicate type III secretion effector heterogeneity within the collection, whereas the high prevalence of *toxA*, *lasB*, *algD*, *pvdS*, and *hcp1* shows that the observed resistance phenotypes occurred in isolates retaining several canonical pathogenicity-associated systems. Because this was not a comprehensive virulome study and no expression, functional, or clinical-severity data were available, these observations remain descriptive and should not be interpreted as evidence of differential pathogenicity [8,14,16].

For clinicians, the data support culture and quantitative susceptibility testing in recurrent, chronic, or previously treated pseudomonal otitis. Empirical fluoroquinolone use is difficult to justify in a collection with such a high resistant proportion under current canine breakpoints. Aminoglycosides retained favorable in vitro activity in most isolates, but treatment selection must still account for tympanic membrane integrity, ototoxicity risk, formulation, and the possibility of otitis media. Topical drug concentrations can exceed MICs, yet this should not be used to dismiss susceptibility testing because biofilm, exudate, and poor distribution can markedly reduce effective exposure [2,8,53].

This study has several limitations. The isolates represent an archival convenience collection with small sample size from two discrete years rather than a prospective population-based survey. Clinical metadata, prior antimicrobial exposure, disease severity, laterality, chronicity, and treatment outcome were unavailable. The analytical panel included compounds without validated canine interpretive criteria, necessitating descriptive or ECOFF-based reporting. Eight phenotyped isolates lacked matched genomic results, and 6 of the 59 assemblies did not meet the prespecified CheckM quality threshold. The use of long-read-only assemblies can retain residual base-level errors even after polishing, which may affect allele calls. The resistance-determinant analysis focused primarily on gene presence and did not include systematic QRDR or regulatory-variant calling, promoter analysis, gene-expression profiling, or biofilm phenotyping. Mobile-element and plasmid assignments were computational predictions. The selected virulence-associated loci were reported only as contextual descriptors and do not constitute a comprehensive analysis of virulome architecture, gene-content diversity, or functional virulence. Finally, the two sampling years do not support continuous trend modelling. These constraints were addressed by conservative interpretation, explicit separation of clinical breakpoints from ECOFFs, false-discovery-rate control, and avoidance of causal or transmission claims.

Nevertheless, this study provides a historical baseline for canine otitis-associated *P. aeruginosa* in Hungary. It complements recent contemporary datasets from Europe and elsewhere [3,5,7,8,9,10,43,54,55,56], documents the effect of applying modern canine fluoroquinolone breakpoints to older isolates, and provides matched quantitative phenotype, resistance-associated genomic, mobile-context, and selected contextual-marker information. Such historical collections are particularly useful when interpreted transparently because they allow future work to distinguish true temporal change from differences introduced by methods or breakpoints.

## 5. Conclusions

Archival canine otitis-associated *P. aeruginosa* isolates collected in Hungary in 2010 and 2017 showed high fluoroquinolone non-susceptibility, generally low aminoglycoside and ceftazidime MICs, and a frequently non-wild-type piperacillin–tazobactam distribution. Their genomes carried a broad and largely conserved intrinsic resistance backbone. Variable determinants, including *crpP* and PDC/OXA alleles, did not robustly explain quantitative MIC variation after correction for multiple testing. These findings demonstrate that resistance-gene detection and MIC testing answer different questions: genomics defines mechanistic potential and epidemiological context, whereas standardized phenotyping remains essential for therapeutic interpretation. Continued integration of both approaches, supported by rigorous breakpoint selection and targeted validation of potentially mobile loci, is necessary for antimicrobial stewardship and surveillance of canine pseudomonal otitis.

## Figures and Tables

**Table 1:** Minimum inhibitory concentration (MIC) distributions of the 67 archival canine otitis-associated *Pseudomonas aeruginosa* isolates.

Agent	Abbr.	Min	Max	MIC_50_	MIC_90_	2010 MIC_50_	2010 MIC_90_	2017 MIC_50_	2017 MIC_90_
Enrofloxacin	ENR	0.06	32.0	0.5	4.0	0.5	4.0	0.5	4.0
Marbofloxacin	MAR	0.001	8.0	0.5	2.0	1.0	2.0	0.25	2.0
Ciprofloxacin	CIP	0.004	8.0	0.06	1.0	0.06	0.5	0.06	1.0
Norfloxacin	NOR	0.004	32.0	0.25	4.0	0.25	8.0	0.25	4.0
Orbifloxacin	ORB	0.25	256.0	2.0	16.0	4.0	16.0	2.0	8.0
Tobramycin	TOB	0.125	16.0	0.25	1.0	0.25	4.0	0.25	0.5
Gentamicin	GEN	0.125	4.0	0.5	1.0	0.5	1.0	0.5	0.5
Amikacin	AMK	0.008	8.0	0.5	1.0	0.5	1.0	0.5	2.0
Ceftazidime	CAZ	0.03	8.0	0.5	2.0	0.5	2.0	0.5	4.0
Cefoperazone	CFP	0.125	32.0	4.0	8.0	4.0	16.0	2.0	8.0
Chlorhexidine	CHX	1.0	32.0	8.0	16.0	8.0	16.0	8.0	16.0
Imipenem	IPM	0.125	8.0	2.0	4.0	2.0	4.0	2.0	4.0
Polymyxin B	PMB	0.5	64.0	1.0	8.0	2.0	32.0	1.0	4.0
Piperacillin–tazobactam	TZP	4.0	256.0	32.0	64.0	32.0	64.0	32.0	64.0

MIC values are expressed in µg/mL. MIC_50_ and MIC_90_ are the lowest tested concentrations inhibiting at least 50% and 90% of isolates, respectively.

**Table 2:** Interpretive classification using current canine Clinical and Laboratory Standards Institute (CLSI) breakpoints or European Committee on Antimicrobial Susceptibility Testing (EUCAST) epidemiological cut-off values.

Agent	Framework	Lower Category	Intermediate Category	Upper Category
Enrofloxacin	CLSI canine	S 1 (1.5%)	SDD 7 (10.4%)	R 59 (88.1%)
Marbofloxacin	CLSI canine	S 5 (7.5%)	SDD 19 (28.4%)	R 43 (64.2%)
Ciprofloxacin	EUCAST ECOFF	WT 60 (89.6%)	—	NWT 7 (10.4%)
Tobramycin	EUCAST ECOFF	WT 63 (94.0%)	—	NWT 4 (6.0%)
Gentamicin	EUCAST ECOFF	WT 67 (100%)	—	NWT 0
Amikacin	EUCAST ECOFF	WT 67 (100%)	—	NWT 0
Ceftazidime	EUCAST ECOFF	WT 67 (100%)	—	NWT 0
Imipenem	EUCAST ECOFF	WT 66 (98.5%)	—	NWT 1 (1.5%)
Piperacillin–tazobactam	EUCAST ECOFF	WT 16 (23.9%)	—	NWT 51 (76.1%)

S, susceptible; SDD, susceptible dose-dependent; R, resistant; WT, wild type; NWT, non-wild type; ECOFF, epidemiological cut-off value. ECOFF categories describe population biology and are not veterinary clinical breakpoints.

**Table 3:** Selected resistance-associated genomic features in the matched isolate set (*n* = 59).

Feature	*n*/59	Interpretive Role
MexAB–OprM efflux system	58	Broad intrinsic efflux background
MexCD–OprJ efflux system	57	β-lactam and multidrug efflux
MexEF–OprN efflux system	58	Fluoroquinolone-associated efflux
MexXY–OprM efflux system	58	Aminoglycoside-associated efflux
PDC β-lactamase allele	58	Chromosomal AmpC-family β-lactamase
OXA-50-like β-lactamase allele	58	Intrinsic class D β-lactamase family
*APH(3′)-IIb*	58	Aminoglycoside-modifying enzyme
*arnA*	58	Lipid A modification pathway
*basS*	57	Two-component regulatory system
*crpP*	37	Fluoroquinolone-modifying determinant
Plasmid-predicted *crpP*	6	Predicted plasmid-like contig

## Data Availability

The whole-genome assemblies generated in this study are openly available in NCBI under BioProject PRJNA1499158 at https://www.ncbi.nlm.nih.gov/bioproject/PRJNA1499158, reference number PRJNA1499158. The corresponding isolate-level BioSample accessions are provided in Supplementary Table S1. Isolate-level phenotypic susceptibility data, genome-quality metrics, and selected genomic markers are provided in Table S1, while complete correlation, determinant-level, and sensitivity-analysis outputs are provided in Tables S2–S4. Genomic context information for the PlasFlow-predicted *crpP* loci is provided in Table S5. Sample Python code supporting reproducibility of the principal statistical, phenotype–genotype, sensitivity, and mobile-element-context analyses is publicly available at https://github.com/kerekadam03/canine-otitis-pseudomonas-analysis (accessed on 18 August 2026).

## Supplementary Materials

The following supporting information can be downloaded at https://www.mdpi.com/article/10.3390/vetsci13090855/s1: Table S1. Isolate-level phenotypic susceptibility data, genome-quality metrics, and selected genomic markers for the archival canine otitis-associated *Pseudomonas aeruginosa* collection; Table S2. Spearman rank correlations between total CARD resistance-associated determinant counts and log_2_-transformed MIC values in the matched isolate set; Table S3. Associations between selected resistance-associated determinants and MIC distributions in the matched isolate set; Table S4. Sensitivity analyses restricted to the 53 assemblies meeting the predefined CheckM quality criteria; Table S5. Genomic context of PlasFlow-predicted *crpP* loci.
